# Portable Molecular Diagnostics for Cetacean Morbillivirus: Development of a Reverse Transcription Insulated Isothermal PCR (RT‐iiPCR) for Global Surveillance

**DOI:** 10.1155/tbed/4194454

**Published:** 2026-02-23

**Authors:** Chricel Lattao, Cheng-Chen Hsieh, Marie Christine M. Obusan, Antonio Fernández, Kristi West, Kátia R. Groch, José Luiz Catão-Dias, Kang-Ning Shen, Wei-Cheng Yang

**Affiliations:** ^1^ Institute of Biology, College of Science, University of the Philippines Diliman, Quezon City, Philippines, upd.edu.ph; ^2^ School of Veterinary Medicine, National Taiwan University, Taipei, Taiwan, ntu.edu.tw; ^3^ Institute for Animal Health and Food Safety, University of Las Palmas de Gran Canaria, Las Palmas de Gran Canaria, Canary Islands, Spain, ulpgc.es; ^4^ Health and Stranding Lab, Human Nutrition, Food and Animal Sciences, College of Tropical Agriculture and Human Resilience, University of Hawaii at Manoa, Honolulu, Hawaii, USA, hawaii.edu; ^5^ School of Veterinary Medicine and Animal Sciences, University of São Paulo, São Paulo, Brazil, usp.br; ^6^ Marine Ecology and Conservation Research Center, National Academy of Marine Research, Kaohsiung, Taiwan

**Keywords:** cetaceans, disease, epidemiology, *Morbillivirus*, surveillance, unusual mortality event

## Abstract

Cetacean morbillivirus (CeMV) drives recurrent unusual‐mortality events, yet surveillance is uneven where laboratory capacity is limited. We developed a portable reverse transcription‐insulated isothermal PCR (RT‐iiPCR) assay targeting a conserved phosphoprotein (P)‐gene segment and evaluated analytical performance, tissue‐level clinical sensitivity, and concordance with reverse transcription‐quantitative PCR (RT‐qPCR) under low‐copy conditions that resemble challenging strandings. Using synthetic RNA, RT‐iiPCR achieved 100% detection from 62,560 to 513 copies µL^−1^, and probit analysis estimated a 95% limit of detection (LOD_95_) of 139 copies µL^−1^. Clinical sensitivity was assessed with two spiking regimes (RNA added after or before extraction) in cerebrum and lung. Singleplex RT‐iiPCR maintained 100% positivity to approximately cycle threshold (Ct) 33 irrespective of spiking order, indicating that near‐limit failures are governed by template scarcity rather than extraction loss. Duplex RT‐iiPCR co‐amplifying β2‐microglobulin (B2M) provided process control with a slight sensitivity cost, sustaining perfect detection to ~Ct 30–31. Across low‐copy panels, agreement with RT‐qPCR was substantial (overall *κ* = 0.68–0.76) and very good in cerebrum (singleplex *κ* = 0.85; duplex *κ* = 0.87), while duplex lung showed lower concordance (*κ* = 0.55) driven solely by Ct >33 false‐negative calls, with no false positives. The assay detected five lineages (dolphin morbillivirus [DMV], pilot whale morbillivirus [PWMV], beaked whale morbillivirus [BWMV], Guiana DMV [GDMV], and Fraser’s DMV [FDMV]) in formalin‐fixed, paraffin‐embedded tissues archived up to 28 years, and sequence alignments indicate expected coverage of additional lineages. Lyophilized reagents, compact hardware, and a quick, simple workflow support deployment in hot, humid, resource‐limited settings. A strain‐agnostic, field‐ready RT‐iiPCR can underpin transboundary CeMV surveillance, enable rapid carcass triage and sequencing, and provide early warning where diagnostic gaps currently exist.

## 1. Introduction

Cetacean morbillivirus (CeMV) is a linear, nonsegmented, negative‐sense single‐stranded RNA (‐ssRNA) virus, classified within the family Paramyxoviridae and the genus *Morbillivirus*. The genus *Morbillivirus* also comprises several other important viruses, including measles virus (MV) and canine distemper virus (CDV) [1–3]. There are eight known strains of CeMV that are neither species‐ nor location‐specific [4]. This includes dolphin morbillivirus (DMV) [5], porpoise morbillivirus (PMV) [6], pilot whale morbillivirus (PWMV) [7], beaked whale morbillivirus (BWMV) [8], a strain from Indo‐Pacific bottlenose dolphins (*Tursiops aduncus*) in Western Australia [9], Guiana DMV (GDMV) [10], DMV Northeast Atlantic (NE‐Atlantic) [11], and Fraser’s dolphin morbillivirus (FDMV) [12]. Similar to other morbilliviruses, CeMV transmission primarily occurs via inhalation of aerosolized viral particles, vertical transmission, and possibly fecal shedding [13–15]. Infection can cause acute to chronic systemic diseases and persistent localized encephalitis, significantly affecting cetacean buoyancy and mobility, thus increasing stranding and mortality risks (reviewed in [16]). Lifelong immunity develops postinfection, leaving immunologically naïve populations vulnerable to novel strains and unusual mortality events (UMEs) [17, 18]. CeMV has been reported in other marine mammals like pinnipeds [19, 20] and Eurasian otters (*Lutra lutra*) [21]. This raises concerns about its potential for cross‐species transmission, which may disrupt marine ecosystem stability and increase the risk of emerging zoonotic threats. CeMV is one of the major cetacean emerging and re‐emerging pathogens (EREPs) that can cause epidemics and UMEs [22].

Classical assays such as virus isolation, antigen‐ or antibody‐ELISAs, virus neutralization, immunohistochemistry, immunofluorescence, and electron microscopy remain the cornerstone for confirming infection, characterizing live virus, and establishing sero‐epidemiological baselines. These methods, however, depend on cell‐culture infrastructure, reference antisera, or advanced imaging equipment and often require days to weeks to deliver results [12, 23–25]. Molecular diagnostics, including conventional and quantitative RT‐PCR, provide superior sensitivity and enable rapid genotyping via sequencing or high‐resolution melting [26–28]. Despite these advantages, they still rely on benchtop thermocyclers, cold‐chain reagents, and skilled personnel, requirements that are often unavailable in many coastal stranding‐response facilities and resource‐limited regional laboratories. In addition, these methods are time‐consuming, often requiring more than half a day to complete the process from sample preparation to results. Collectively, these hurdles delay timely diagnosis and perpetuate uneven global surveillance.

These diagnostic constraints help explain the apparent patchiness of CeMV reports outside of well‐resourced regions. Although CeMV is globally distributed, most documented outbreaks originate from the Atlantic Ocean and Mediterranean Sea, where robust diagnostic capacity exists [22]. Three decades of Mediterranean UMEs demonstrate how robust surveillance can reveal sustained CeMV circulation when diagnostic capacity is in place. These events include the 1990 mass mortality of roughly 1000 striped dolphins, a 2006–2007 event involving more than 100 individuals, and recent viral activity along the Italian coast between 2018 and 2021 [22, 29, 30]. Conversely, reports from the Western Pacific and Indian Oceans remain sporadic, being largely confined to isolated cases in Japan, Taiwan, and the Philippines, despite ecological indications that CeMV circulates more widely in these basins [31–33]. This apparent scarcity is best explained by a nexus of practical constraints: severe weather and remote shorelines that delay carcass recovery; long‐distance transport in warm climates and limited access to proper storage and molecular laboratories that accelerate RNA degradation; and, in several nations, an absence of a designated agency to coordinate stranding case diagnostics. Together with chronic underfunding, these factors hamper systematic sampling and underscore the urgency of portable, field‐ready diagnostics capable of delivering reliable results close to the point of stranding [15, 34, 35].

Effective CeMV surveillance requires a test that captures the widest possible strain diversity, yields an amplicon amenable to sequencing, and produces results quickly enough for carcass triage, thereby enabling prompt and affordable epidemiological assessment. Rapid on‐site screening enables positive animals to be prioritized for detailed pathology and full genomic characterization. Moreover, wider geographical coverage ensures that prevalence estimates can be integrated into transmission models and management plans. Closing these monitoring gaps is essential for timely responses to emerging CeMV outbreaks. To meet these needs, we designed a reverse transcription‐insulated isothermal PCR (RT‐iiPCR) assay targeting a conserved segment of the phosphoprotein (P)‐gene. The assay runs on a portable iiPCR device with an automatic nucleic acid extraction device as a rapid, affordable, user‐friendly, and field‐deployable platform. The assay was validated on a panel of CeMV strains spanning the recognized genetic lineages.

## 2. Materials and Methods

### 2.1. Ethics Statement

All animal procedures were conducted with the approval of the Ocean Conservation Administration (Permit #090002352) and the National Academy of Marine Research (NAMR‐114‐036), Ocean Affairs Council, Taiwan.

### 2.2. CeMV DNA and RNA Template

Initial testing of the primers and probe was conducted using a DNA template (gBlocks Gene Fragments) and an RNA template (Ultramer RNA Oligo). The DNA template for the initial trial was synthesized based on a 283‐bp fragment, while the RNA template was derived from an 80‐bp region of the P gene sequence of the DMV strain (GenBank accession no. AJ608288.1) and the BWMV strain (GenBank accession no. KM460045.1). A 100 µM RNA template was prepared by dissolving 4 nmol of synthesized RNA in a 40 µL mixture consisting of 38 µL RNA storage solution (Invitrogen) and 2 µL RNase inhibitor (Applied Biosystems). This stock was then used to prepare a range of diluted RNA samples, which were stored at −80°C until use.

### 2.3. Primer and Probe Design

A reverse primer and a probe were newly designed based on alignments of P gene sequences of CeMV strains from GenBank using MEGA v.11 [36], while the forward primer was adopted from Barrett et al. [2] (Table 1). The reverse primer and probe were designed according to the recommendations provided by the iiPCR manufacturer (GeneReach, Taiwan). The design parameters included a primer melting temperature (*T*_*m*_) of 58–62°C, a GC content between 20% and 80%, and a primer length of 15–25 bp. The amplicon length was kept below 150 bp. The probe was positioned at least one base away from the primers, with a *T*_*m*_ ~10°C higher (68–72°C) than that of the primers. Primer‐BLAST was used to check the properties of the primer. Primers and probes targeting the β2‐microglobulin (B2M) gene were designed based on the sequence of the bottlenose dolphin (*Tursiops truncatus*) (GenBank accession no. DQ404542.1) [35, 37, 38]. B2M was used as an internal control in duplex clinical sensitivity tests to ensure that negative results were not caused by poor sample quality or errors during tissue preparation and nucleic acid extraction. The fluorescent‐labeled probe targeting the P gene was labeled with FAM (emission at 520 nm), while the probe for B2M was labeled with VIC (emission at 550 nm) (Table 1).

**Table 1:** Primers and probes sequences.

Primers and probes	Nucleotide sequence (5′‐3′)	Target genes	Function	Amplicon (bp)	References
CeMV_F	5′‐ATGTTTATGATCACRGCGGT‐3′	P gene	Phosphoprotein	70	Barrett et al. [2]
CeMV_R	5′‐CCTGTTGGAACCACGAG‐3′				This study
CeMV_Probe	5′‐6‐FAM‐GAGTCAAGGATGCTGAC‐MGB‐NFQ‐3′				
B2M_F	5′‐GGTGGAGCAATCAGACCTGT‐3′	B2M gene	𝛽2‐microglobulin	78	Chen et al. [37], [38])
B2M_R	5′‐GCGTTGGGAGTGAACTCAG‐3′				
B2M_Probe	5′‐VIC‐TCAGCAAGGACTGGTCTT‐MGB‐NFQ‐3′				

### 2.4. Nucleic Acid Preparation

The nucleic acids were extracted from the cerebrum and lung tissues of a validated CeMV‐negative stranded Fraser’s dolphin (*Lagenodelphis hosei*) found in Taiwan in 2023, using the Taco mini magnetic bead‐based nucleic acid extraction system (GeneReach, Lexington, MA, USA) (Figure 1). The clinical sensitivity of the RT‐iiPCR assay was evaluated by spiking serially diluted CeMV RNA templates into the nucleic acids extracted from tissue samples. Approximately 25 mg of tissue was homogenized in 250 µL of nuclease‐free water (NFW; Qiagen), followed by centrifugation for 5 min. A volume of 200 µL of the resulting supernatant was transferred to the first well of the extraction plate containing lysis buffer. Nucleic acid extraction was performed according to the manufacturer’s instructions. For each reaction, 10 µL of the extracted nucleic acid was mixed with 40 µL of CeMV RNA template and 1.25 µL of RNase inhibitor.

Figure 1Schematic diagram of the detection workflow and portable field‐deployable platform for CeMV using RT‐iiPCR. (A) Following organ collection (e.g., lung and brain) from cetaceans, ~25 mg of tissue is homogenized and centrifuged, and the resulting supernatant is used for nucleic acid extraction. A 5 µL aliquot of the extracted nucleic acid is added to the reconstituted premix and transferred into an R‐tube, which is then placed into the dual‐channel POCKIT Nucleic Acid Analyzer (GeneReach), capable of detecting fluorescence at 520 and 550 nm. The system allows for eight reactions per run, with each run lasting ~58 min. Fluorescent signals generated through probe hydrolysis during amplification are analyzed by the instrument’s built‐in algorithm, and results are displayed as readable signals (“+”, “−”, or “?”). When 550 nm (B2M) shows “+”, it indicates successful nucleic acid extraction, and the results can be considered reliable. At this point, a 520 nm (CeMV) reading of “+” indicates a positive result, “‐” indicates a negative result, and “?” indicates that the viral load may be near the detection limit. When 550 nm (B2M) shows “‐”, whiich indicates poor nucleic acid extraction quality, and the results should not be considered reliable. A warning sign “!” may also appear, which indicates the need to prepare and run a new reaction in the same well. The diagram is adapted from Hsieh and Yang [35] and Carossino et al. [39]. (B) The entire testing kit, including the POCKIT Nucleic Acid Analyzer (100–120/200–240 V AC), Taco mini magnetic bead‐based nucleic acid extraction system (100–120V/200–240 V AC), and necessary reagents, is housed in a rugged protective case designed for field transport and on‐site molecular diagnostics. Consumables needed for testing, include microcentrifuge tubes, pipette tips, pellet pestle, Taco Extraction kit, POCKIT Enzyme/dNTP with 2x Premix Buffer B Set, R‐tube, and nuclease‐free water. Additional equipment, like a spin‐down microcentrifuge, may also be included in this kit. Image adapted from GeneReach Biotechnology Corp. [40].

**Figure:**
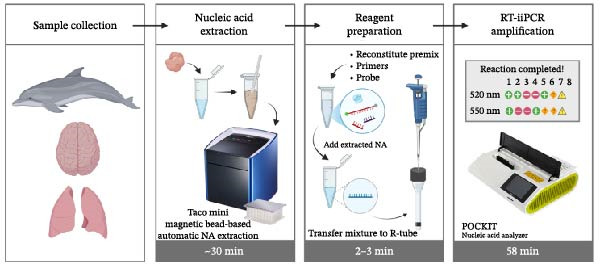
(A)

**Figure:**
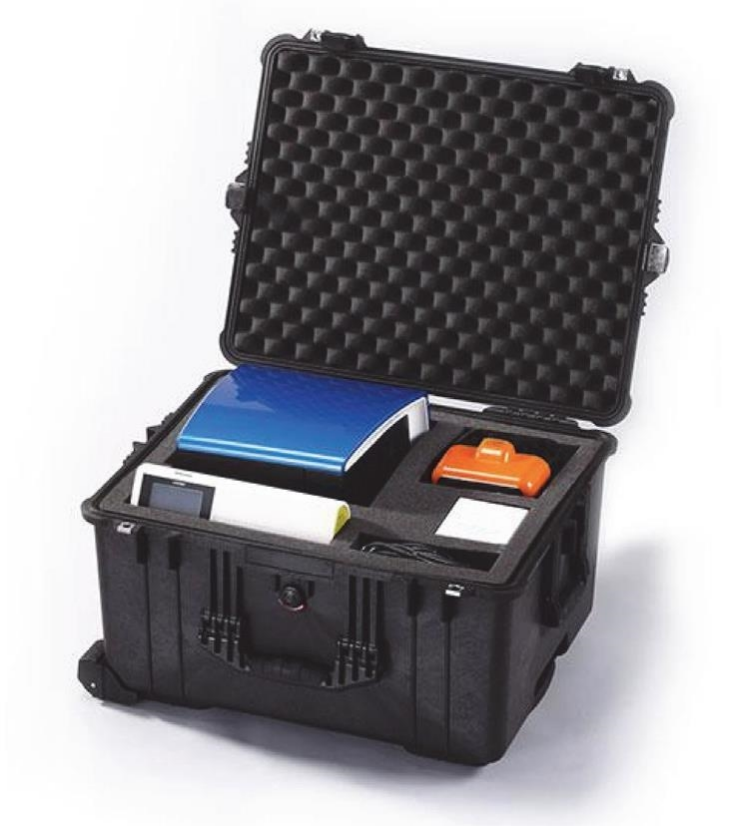
(B)

A further validation of the RT‐iiPCR test using tissue samples was performed by spiking serially diluted CeMV RNA templates into cerebrum and lung tissues prior to nucleic acid extraction. Approximately 25 mg of each tissue sample was submerged in RNAlater Stabilization Solution (Invitrogen) and incubated overnight at 4°C, then stored at −20°C until extraction. For each test, 20 µL of CeMV RNA template and 250 µL of NFW were added to the tissues, followed by homogenization on ice. The homogenate was centrifuged for 5 min, and 200 µL of the supernatant was subjected to nucleic acid extraction using the same protocol as the clinical sensitivity assay. A total of 200 µL of extracted nucleic acid was obtained. For each trial, 80 µL of reaction mixture, comprising 78 µL of extracted nucleic acid and 2 µL of RNase inhibitor, was prepared for each RNA concentration.

To evaluate the applicability of the developed protocol to actual clinical samples, 12 CeMV‐positive formalin‐fixed paraffin‐embedded (FFPE) tissue blocks were tested using RT‐iiPCR. These samples, representing four different CeMV strains, were collected from stranded cetaceans in Spain (PWMV/DMV), Brazil (GDMV), and Hawaii (BWMV/FDMV) between 1996 and 2018 that were obtained from previous studies [28]. The FDMV strain originated from samples collected in Hawaii in 2018 under CITES Permit No. OAC3C114030011. Additionally, two FFPE samples from a short‐finned pilot whale (*Globicephala macrorhynchus*) stranded in Taiwan in 2024 were included in the analysis (Table 2). Total RNA was extracted from two tissue sections (8 µm thick) per FFPE block using the RNeasy FFPE Kit (Qiagen).

**Table 2:** Clinical validation using FFPE blocks from multiple geographic regions, including archived CeMV‐positive and unconfirmed specimens.

Species	Years	Countries	Strains	Organs	P gene/B2M RT‐iiPCR (no. of positive/no. of tested)	P gene/B2M RT‐qPCR (no. of positive/no. of tested) (Cq value)	References
*Globicephala macrorhynchus*	1996	Canary Is., Spain	PWMV	Cerebrum	2/2	2/2 (28.36)	Belliere et al. [41]
*Sotalia guianensis*	2010	Brazil	GDMV	Lymph node	2/2^a^	2/2 (32.06)	Groch et al. [10]
*Indopacetus pacificus*	2010	Hawaii	BWMV	Lymph node	2/2	2/2 (27.69)	West et al. [8]
*Stenella coeruleoalba*	2008	Canary Is., Spain	DMV	Cerebrum	2/2	2/2 (29.28)	Sierra et al. [42]
*Stenella coeruleoalba*	2011	Canary Is., Spain	DMV	Cerebrum	2/2	2/2 (29.09)	
*Tursiops truncatus*	2005	Canary Is., Spain	DMV	Lymph node	2/2	2/2 (23.32)	[43]
*Globicephala melas*	2007	Spain	DMV	Cerebrum	2/2	2/2 (32.49)	Fernández et al. [14]
				Lymph node	2/2	2/2 (23.39)	
				Lung	2/2	2/2 (24.49)	
*Lagenodelphis hosei*	2018	Hawaii	FDMV	Cerebellum	2/2	2/2 (29.84)	West et al. [12]
				Lymph node	2/2	2/2 (28.99)	
				Lung	2/2	2/2 (31.69)	
*Globicephala macrorhynchus*	2024	Taiwan	—	Cerebrum	0/4^b^	0/4	This study
				Lymph node	0/4^b^	0/4	

^a^Positive P gene detection but negative B2M results.

^b^Negative P gene detection despite positive B2M results.

### 2.5. RT‐iiPCR

Reaction mixture preparation varied by assay type. For initial primer and probe evaluation, analytical sensitivity, and singleplex clinical sensitivity tests, a 45 µL reaction mixture was prepared containing 2.5 µL each of 10 µM forward and reverse primers, 1.5 µL of 5 µM probe, 25 µL of 2× Premix Buffer B (POCKIT Enzyme/dNTP with 2X Premix Buffer B Set, GeneReach), and 13.5 µL of NFW. For duplex clinical sensitivity tests incorporating the B2M internal control, the 45 µL reaction mixture included P gene components (2.5 µL each of 10 µM forward and reverse primers, 1.5 µL of 5 µM probe), B2M gene components (1 µL each of 10 µM forward and reverse primers, 0.375 µL of 5 µM probe), 25 µL of 2× Premix Buffer B, and 11.125 µL of NFW. All reaction mixtures were added to Enzyme/dNTP tubes (POCKIT Enzyme/dNTP with 2X Premix Buffer B Set, GeneReach) containing lyophilized pellets of dNTPs, reverse transcriptase, and amplification enzymes.

For analytical sensitivity tests, 5 µL of pure RNA templates was added to the reaction mixture. For singleplex and duplex clinical sensitivity tests, 5 µL of spiked nucleic acids was used instead. A total of 50 µL of the prepared reaction solution was transferred to an R‐tube (GeneReach), capped, and briefly centrifuged for 10 s. The tubes were then placed into the dual‐channel POCKIT Nucleic Acid Analyzer (GeneReach), capable of detecting fluorescence at 520 nm and 550 nm. The reaction time was ~58 min, after which results were automatically interpreted and displayed as “+,” “−,” or “?” based on the signal‐to‐noise (S/N) ratio (Figure 1).

### 2.6. Reverse Transcription‐Quantitative PCR (RT‐qPCR)

All samples were tested using both RT‐iiPCR and RT‐qPCR for comparison. Template types varied by assay: pure RNA templates were used for analytical sensitivity tests, while extracted nucleic acids spiked with RNA templates were used for clinical sensitivity tests. RT‐qPCR reaction mixtures were prepared according to assay format. For analytical sensitivity and singleplex testing, the 20 µL reaction mixture contained 1 µL each of 10 µM forward and reverse primers, 1 µL of 5 µM probe, 0.1 µL of RT enzyme solution, 4 µL of RT‐qPCR master mix (LightCycler Multiplex RNA Virus Master), 7.9 µL of NFW, and 5 µL of template. For duplex RT‐qPCR targeting both the P gene and B2M, the 20 µL reaction mixture consisted of 1 µL each of 10 µM primers and 1 µL of 5 µM probe for both targets, 0.1 µL of RT enzyme solution, 4 µL of RT‐qPCR master mix (LightCycler Multiplex RNA Virus Master), 4.9 µL of NFW, and 5 µL of extracted nucleic acid.

RT‐qPCR was performed on a QuantStudio 3 Real‐Time PCR System (Applied Biosystems) using fast mode and TaqMan probe settings. The thermal profile consisted of reverse transcription at 50°C for 10 min, preincubation at 95°C for 30 s, followed by 40 cycles of two‐step amplification (95°C for 5 s, 60°C for 30 s), and a final hold at 40°C for 30 s. For DNA template qPCR, the 20 µL reaction mixture included 0.8 µL each of 10 µM primers and 5 µM probe, 10 µL of QuantiNova Probe PCR Master Mix (Qiagen), 2.6 µL of NFW, and 5 µL of DNA template. The thermal profile was modified to initial activation at 95°C for 2 min, followed by 40 cycles of denaturation at 95°C for 5 s and annealing/extension at 60°C for 5 s. All reactions were run in triplicate with negative controls (NFW). Raw cycle threshold (Ct) values generated by RT‐qPCR were converted to genome‐copy equivalents per reaction with a standard‐curve equation (*y* = −3.24*x* + 38.87; *R* ^2^ = 0.996, efficiency = 103.54%) derived from the same dilution series (NEB qPCR calculator; https://nebiocalculator.neb.com/#!/qPCRGen). Concentrations are therefore reported throughout this report as calculated copy numbers rather than the prepared dilution factor to provide a direct, cross‐platform measure of template abundance. Samples with Ct values >38.67 were considered negative.

### 2.7. Statistics

Statistical analysis was performed to evaluate assay performance and concordance. The 95% limit of detection (LOD_95_) was estimated using probit analysis implemented via the glm function in R Studio (Version 2024.04.2). Agreement between RT‐iiPCR and RT‐qPCR results for both singleplex and duplex tests was assessed using Cohen’s kappa coefficient, calculated from 2 × 2 contingency tables (http://vassarstats.net/kappa.html).

## 3. Results

**Figure 2:**
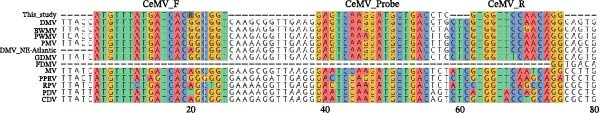
Sequence alignment of CeMV strains with the primer pair and probe. Multiple sequence alignment was done using MEGA v.11 [36]. This figure was made using the visualization tool “ggmsa” in R [44]. The following are the accession numbers of each sequence: DMV (AJ608288.1), BWMV (KM460045.1), PWMV (ON513035.1), PMV (MH430945.1), DMV‐NE Atlantic (MG000861.1), GDMV (PV298625), FDMV (MZ485915.1), MV (OP236009.1), PPRV (OL310685.1), RPV (JN234010.1), PDV (KC802221.1), and CDV (AF164967.1).

**Figure 3:**
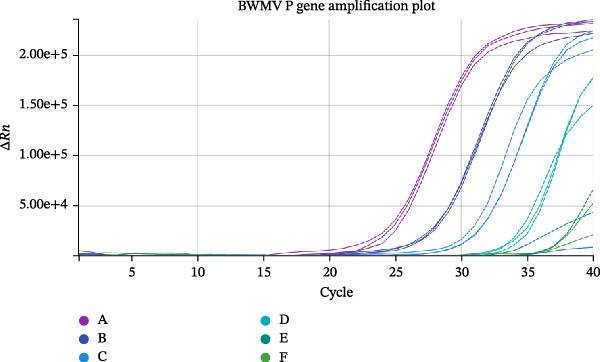
Amplification plot of singleplex analytical sensitivity testing of BWMV template in RT‐qPCR. Each letter is color‐coded and represents the copy numbers (copies/µL): (A) 62,560; (B) 5544; (C) 513; (D) 38; (E) 6; and (F) 2. The detailed results for this test are described in Table 3.

Analytical specificity was confirmed through in silico analysis using Primer‐BLAST. The search, conducted against the NCBI nr database and limited to viral sequences, showed specific matches to various CeMV strains, including DMV, PMV, and PWMV. No matches were identified with other morbilliviruses such as CDV, phocine distemper virus (PDV), or rinderpest virus (RPV). Although alignments were found with peste des petits ruminants virus (PPRV) and MV, these sequences differed from the primers by ≥2 base pairs and were not considered significant (Figure 2). Two CeMV RNA templates were used for assay validation: one derived from the DMV strain for initial primer and probe testing, and another from the BWMV strain for analytical and clinical sensitivity evaluations. The sequences of these strains differ by only a single base pair at the reverse primer binding site (Figure 2). Analytical sensitivity was evaluated using tenfold serial dilutions of the RNA template (Table 3 and Figure 3). RT‐iiPCR achieved 100% detection at concentrations ranging from 62,560 to 513 copies/µL. At the lowest concentration tested (2 copies/µL), both RT‐iiPCR and RT‐qPCR achieved a 25% detection rate (Ct value = 37.90). Based on probit analysis, the LOD_95_ of RT‐iiPCR was estimated to be 139 copies/µL.

**Table 3:** Analytical sensitivity in singleplex RT‐iiPCR.

RNA template (copies/µL)^a^	P gene RT‐iiPCR (no. of positive/no. of tested)	P gene RT‐qPCR (no. of positive/no. of tested)	Ct value
62,560	4/4	4/4	23.33
5544	4/4	4/4	26.74
513	4/4	4/4	30.09
38	3/4	4/4	33.77
6	3/4	4/4	36.29
2	1/4	1/4	37.9

^a^The copy numbers were calculated from the standard‐curve equation described in Section 2.

Clinical sensitivity was assessed using tissue‐spiked samples in both singleplex and duplex formats under two experimentally distinct spiking regimes (Tables 4 and 5). Across both regimes and in both single‐ and duplex‐reaction formats, every sample that yielded a positive signal by RT‐iiPCR was positive by RT‐qPCR, preserving complete concordance between methods. The sensitivity profile of the singleplex assay (Table 4) was largely unchanged by the additional extraction step. Overall, 100% positivity persisted to Ct values of ~33 regardless of spiking sequence. At the extremity of the dilution series, stochastic dropout occurred with similar frequency in both spiking scenarios when Ct values reached the mid‐thirties. This pattern indicates that template abundance, rather than extraction‐associated RNA loss or PCR inhibition, represents the principal determinant of assay failure near the detection limit. These findings confirm that the taco mini extraction protocol does not materially erode RT‐iiPCR sensitivity. In the duplex configuration (Table 5), the CeMV P gene is co‐amplified with B2M to verify extraction and reverse transcription efficiency. The results show that the duplex RT‐iiPCR showed reduced sensitivity compared to the singleplex format. Perfect detection was retained through Ct ≈30–31 under both spiking regimes and for both organ samples.

**Table 4:** Clinical sensitivity in singleplex RT‐iiPCR and RT‐qPCR.

Organs	P gene RT‐iiPCR (no. of positive/no. of tested)	P gene RT‐qPCR (no. of positive/no. of tested)	Ct value^a^	Viral genome copies per reaction (copies/µL)^b^
Cerebrum	1/1	1/1	27.47	3300
	4/4	4/4	29.65	701
	4/4	4/4	32.78	76
	2/3	3/3	34.81	18
	2/2	2/2	27.56	3096
	2/2	2/2	30.09	513
	3/4	4/4	33.45	47
	0/3	2/3	35.17	14
Lung	4/4	4/4	29.96	562
	5/5	5/5	33.27	54
	3/5	5/5	36.07	7
	0/2	1/2	37.28	3
	1/1	1/1	26.96	4742
	4/4	4/4	31.67	167
	3/4	4/4	35.31	13
	1/3	1/3	35.95	10

*Note:* White rows: RNA dilutions spiked into tissue extracts; gray rows: RNA dilutions spiked into tissues before extraction.

^a^Average of Ct values from RT‐qPCR.

^b^The copy numbers were calculated from the standard‐curve equation described in Section 2.

**Table 5:** Clinical sensitivity in duplex RT‐iiPCR and RT‐qPCR.

Organs	P gene RT‐iiPCR (no. of positive/no. of tested)	P gene RT‐qPCR (no. of positive/no. of tested)	Ct value^a^	Viral genome copies per reaction (copies/µL)^b^
Cerebrum	1/1	1/1	27.96	2330
	4/4	4/4	30.85	299
	3/4	4/4	33.99	32
	1/3	1/3	37.43	3
	2/2	2/2	27.01	4576
	2/2	2/2	29.31	893
	1/4	4/4	33.57	43
	0/3	2/3	34.88	17
Lung	4/4	4/4	28.83	1255
	2/5	5/5	33.06	62
	1/5	3/5	35.93	8
	1/1	1/1	27.50	3230
	4/4	4/4	30.25	458
	0/4	4/4	34.32	25
	0/3	3/3	36.76	4

*Note:* White rows: RNA dilutions spiked into tissue extracts; gray rows: RNA dilutions spiked into tissues before extraction.

^a^Average of Ct values from RT‐qPCR.

^b^The copy numbers were calculated from the standard‐curve equation described in Section 2.

Agreement between RT‐iiPCR and RT‐qPCR was assessed using 37 samples each of RNA templates spiked into cerebrum and lung tissue extracts. All test samples contained relatively low viral concentrations (Ct values >27), which approach the detection limits of both assays. Overall agreement was higher for singleplex (89%, *κ* = 0.76) compared to duplex testing (84%, *κ* = 0.68), with both demonstrating substantial agreement according to standard kappa interpretation guidelines. No false positives were observed, with overall agreement being higher for singleplex testing (89%, *κ* = 0.76) than duplex testing (84%, *κ* = 0.68), and both results indicate substantial agreement following standard kappa interpretation guidelines (Tables 6, 7). Tissue‐specific performance revealed notable differences in assay concordance. Cerebrum samples showed consistently high agreement in both singleplex (94%, *κ* = 0.85 [0.56–1.00]) and duplex formats (94%, *κ* = 0.87 [0.63–1.00]). In contrast, lung samples demonstrated greater variability, with singleplex achieving 86% agreement (*κ* = 0.70 [0.39–0.99]) compared to 76% for duplex (*κ* = 0.55 [0.24–0.86]).

**Table 6:** Performance evaluation of singleplex RT‐iiPCR.

Organs	P gene RT‐iiPCR	P gene RT‐qPCR			Agreement (*κ*, 95% CI)^a^
		Positive	Negative	Total	
Overall performance	Positive	23	0	23	89% (0.76 [0.54–0.98])
	Negative	4	10	14	
	Total	27	10	37	
Cerebrum	Positive	11	0	11	94% (0.85 [0.56–1.00])
	Negative	1	4	5	
	Total	12	4	16	
Lung	Positive	12	0	12	86% (0.70 [0.39–0.99])
	Negative	3	6	9	
	Total	15	6	21	

^a^The kappa statistic and confidence intervals are shown within brackets.

**Table 7:** Performance evaluation of duplex RT‐iiPCR.

Organs	P gene/B2M RT‐iiPCR	P gene/B2M RT‐qPCR			Agreement (*κ*, 95% CI)^a^
		Positive	Negative	Total	
Overall performance	Positive	16	0	16	84% (0.68 [0.46–0.90])
	Negative	6	15	21	
	Total	22	15	37	
Cerebrum	Positive	9	0	9	94% (0.87 [0.63–1.00])
	Negative	1	6	7	
	Total	10	6	16	
Lung	Positive	7	0	7	76% (0.55 [0.24–0.86])
	Negative	5	9	14	
	Total	12	9	21	

^a^The kappa statistic and confidence intervals are shown within brackets.

Clinical validation was conducted using archived CeMV‐positive FFPE tissues from multiple geographic regions, including the Mediterranean Sea, Atlantic, and Pacific regions (1996–2018) (Table 2). The RT‐iiPCR protocol successfully detected both the P gene and B2M in all CeMV‐positive FFPE samples, with one notable exception: a lymph node sample from a Guiana dolphin (*Sotalia guianensis*) showed positive P gene detection but negative B2M results. RT‐qPCR validation confirmed the reliability of the archived samples. The Ct values obtained were comparable to those previously reported by Yang et al. [28] for the same FFPE sample set, indicating consistent viral RNA preservation and extraction efficiency across different analytical timepoints. The short‐finned pilot whale (*Globicephala macrorhynchus*), known to be susceptible to CeMV [45], was included in this study using FFPE samples from a stranded individual in Taiwan. The tests yielded negative results for the P gene, while the B2M gene was successfully detected, confirming sample integrity (Table 2).

## 4. Discussion

CeMV remains one of the most consequential EREPs in marine mammal medicine, driving repeated UMEs that have left documented demographic impacts on several regional populations [22]. Robust surveillance is therefore a scientific and conservation priority. However, coastal stranding networks outside a handful of well‐resourced regions often lack the laboratory infrastructure needed for molecular confirmation. This study reports on a portable, rapid, and affordable RT‐iiPCR assay that directly addresses these constraints by coupling broad strain coverage with field‐ready deployment. The following discussion benchmarks the assay against existing methods, evaluates its operational and epidemiological value, acknowledges limitations, and outlines future directions.

Several RT‐qPCR protocols already detect multiple CeMV lineages, but each is geographically or logistically constrained. Groch et al. [26] designed “PAN” primers that perform well for Atlantic strains (GDMV, PMV, and PWMV), while Rubio‐Guerri et al. [46] introduced a Universal Probe Library assay targeting the F‐gene that is now routine in the Mediterranean. Yang et al. [28] extended coverage by coupling P gene primers with high‐resolution melting, enabling strain discrimination (DMV, PWMV, BWMV, and GDMV). All of these methods depend on bench‐top thermocyclers, cold‐chain reagents, and trained personnel, requirements that limit their usefulness in decentralized stranding networks and low‐resource settings, despite being analytically robust. The RT‐iiPCR workflow presented here addresses these deficiencies using lyophilized reagents and suitcase‐sized hardware while retaining broad phylogenetic reach. Using synthetic templates and archived FFPE tissues, we detected five lineages, including DMV, PWMV, BWMV, GDMV, and the highly divergent FDMV from Hawaiʻi, demonstrating that the selected P gene segment is sufficiently conserved for universal screening. Successful amplification from FFPE blocks stored for up to 28 years underscores the assay’s resilience to partial RNA degradation and its utility for retrospective surveillance. Although the small sample size used for validation is a recognized limitation, it demonstrated detection across diverse strains, supporting the assay’s broad applicability. Sequence alignments indicate that the assay should detect both PMV and the DMV Northeast Atlantic (NE‐Atlantic) sublineage. By extension, the Western Australian strain, which falls within the same clade, is also expected to amplify. Because substantial surveillance gaps remain; however, previously uncharacterized CeMV variants may already be circulating. It should be noted that both emergent and undiscovered lineages could accumulate substitutions within primer or probe binding sites and reduce efficiency despite overall conservation.

The universal, field‐deployable CeMV assay developed in this study has epidemiological implications that extend well beyond laboratory efficiency. Most recorded outbreaks come from regions with well‐equipped laboratories, so vast stretches of some regions, such as Pacific Island nations, Southeast Asian archipelagos, the western Indian Ocean, and the Amazon basin, remain sparsely sampled. This sampling bias limits our understanding of the virus’s complete geographic distribution, temporal lineage evolution, and interoceanic transmission patterns. Evidence from aquatic animal health shows that iiPCR is already recognized as fit‐for‐purpose by the World Organization for Animal Health: the IQ Plus White Spot Syndrome Virus (WSSV) Kit with POCKIT system (Registration Number 20130108) is certified for on‐site pond‐side use by employing lyophilized reagents. It supports iiPCR as a robust platform for hot, humid, resource‐limited settings. We anticipate that iiPCR will allow coastal stranding networks to generate infection data in real‐time without shipping samples abroad and enable geographically unbiased prevalence maps and meta‐analysis of pooled datasets.

The RT‐iiPCR platform described in this study was demonstrably reactive to all recognized lineages, from Atlantic DMV to the divergent FDMV, and the ability to detect diverse strains can be integrated with additional methods for precise strain identification. This supports consistent criteria for recognizing unusual‐mortality events, a goal now hindered by the variety of strain‐specific tests in use. Rapid, on‐site positivity further improves specimen triage for whole‐genome sequencing, enhancing phylogeographic reconstruction of long‐distance dispersal and clarifying whether supposedly regional strains such as FDMV circulate more widely. Field adoption will also expand background‐prevalence baselines, informing risk models for cross‐species spillover to pinnipeds, otters, or other marine mammals within a One‐Health framework. Finally, beyond its technical merits, a strain‐agnostic RT‐iiPCR enables the integrated, transboundary surveillance architecture required to manage an emerging pathogen whose true ocean‐scale footprint is only beginning to be understood.

To our knowledge, this is the first CeMV assay to combine RT‐iiPCR with a duplex internal control: B2M primers and a probe that amplifies a wide range of odontocetes. Inclusion of B2M allows real‐time verification of extraction efficiency and PCR integrity, mitigating the risk of false negatives in autolyzed or inhibitor‐rich field samples. Collectively, these attributes, including portability, universality across known strains, and built‐in quality control, position the duplex RT‐iiPCR as a practical frontline tool for global CeMV surveillance and outbreak response. However, the choice between duplex and singleplex RT‐iiPCR formats entails a balance between sensitivity and operational robustness. Duplex reactions that co‐amplify the β2‐microglobulin internal control result in enhanced reliability. This quality assurance, however, incurs slight reduction in sensitivity in the cerebrum and lung, likely because B2M dominates the reaction and consumes a disproportionate share of reagents when the viral template is scarce. Singleplex testing avoids this penalty and therefore remains the preferred option when maximal sensitivity is paramount,for example, for chronic encephalitic infections or retrospective studies of archival tissues in which viral RNA is supposed to be already scarce, provided that parallel controls or replicate testing are in place to detect false negatives.

Regulatory authorities, including the FDA’s EUA program, require point‐of‐care assays to demonstrate consistent performance across the full viral‐load spectrum, with particular emphasis on samples near the analytical limit. The present comparative evaluation of RT‐iiPCR and RT‐qPCR instead focused solely on low‐copy templates to model two common surveillance contexts: sporadic, possibly low‐titer strandings outside the core outbreak range, and retrospective investigations that rely on RNA‐degraded FFPE tissues. Under these deliberately stringent conditions, the assays exhibited agreement ranging from good (*κ* = 0.68–0.76) to very good (*κ* = 0.85–0.87). The single exception was lung tissue analyzed in duplex format, where concordance fell to *κ* = 0.55. However, this reduction was driven exclusively by false‐negative calls at Ct >33, and no false positives were observed, indicating that specificity was fully retained. Because acute CeMV infections may yield Ct values in the 20 s (≥10^4^–10^5^ genome copies µL^−1^), which is well above our test range, the duplex assay’s modest sensitivity penalty in the lung is unlikely to affect real‐world case detection. Thus, although our validation did not include the high‐titer tier recommended in standard protocols, it provides a rigorous stress test at the epidemiologically most challenging end of the viral‐load continuum and supports the conclusion that RT‐iiPCR will perform at least as well, and probably better, under routine field conditions.

In light of accelerating environmental change, global CeMV monitoring assumes added urgency. Arctic ice loss, marine heatwaves, and altered current systems are already reshaping prey fields and migration routes, raising the likelihood of new contact networks among cetacean populations and, with them, lineage exchange and long‐range viral dispersal that could precipitate the next large‐scale mortality event [16]. The risk is acute for threatened Indo‐Pacific taxa in which CeMV infection has yet to be reported, including the Indo‐Pacific humpback dolphin (*Sousa chinensis*), Māui dolphin (*Cephalorhynchus hectori maui*), and the Irrawaddy dolphin (*Orcaella brevirostris*), whose small, fragmented populations are vulnerable to additional mortality. Concurrently, cumulative anthropogenic stressors and pollution can impair immune competence, increasing susceptibility to infection and the probability of stranding [47]. These considerations argue for coordinated, global CeMV surveillance with particular emphasis on the Pacific, where data remain sparse and where rapid, field‐based diagnostics such as the present RT‐iiPCR can most effectively close critical information gaps.

## Funding

This research was supported by the National Academy of Marine Research, Ocean Affairs Council (Grant NAMR‐114‐036).

## Conflicts of Interest

The authors declare no conflicts of interest.

## Data Availability

The data that support the findings of this study are available from the corresponding author upon reasonable request.
